# A Simple Colorimetric Assay of Bleomycin‐Mediated DNA Cleavage Utilizing Double‐Stranded DNA‐Modified Gold Nanoparticles

**DOI:** 10.1002/cbic.202200451

**Published:** 2022-10-25

**Authors:** Yoshitsugu Akiyama, Kazunori Kimura, Syuuhei Komatsu, Tohru Takarada, Mizuo Maeda, Akihiko Kikuchi

**Affiliations:** ^1^ Katsushika Division Institute of Arts and Sciences Tokyo University of Science 6-3-1 Niijuku 125-8585 Katsushika Tokyo Japan; ^2^ Department of Materials Science and Technology Graduate School of Advanced Engineering Tokyo University of Science 6-3-1 Niijuku 125-8585 Katsushika Tokyo Japan; ^3^ Surface and Interface Science Laboratory RIKEN 2-1 Hirosawa 351-0198 Wako Saitama Japan; ^4^ RIKEN Cluster for Pioneering Research 2-1 Hirosawa 351-0198 Wako Saitama Japan

**Keywords:** bleomycin, colorimetric assay, DNA-modified gold nanoparticles, DNA damaging agents, non-crosslinking aggregation

## Abstract

A colorimetric assay of DNA cleavage by bleomycin (BLM) derivatives was developed utilizing high colloidal stability on double‐stranded (ds) DNA‐modified gold nanoparticles (dsDNA‐AuNPs) possessing a cleavage site. The assay was performed using dsDNA‐AuNPs treated with inactive BLM or activated BLM (Fe(II)⋅BLM). A 10‐min exposure in dsDNA‐AuNPs with inactive BLM treatment resulted in a rapid color change from red to purple because of salt‐induced non‐crosslinking aggregation of dsDNA‐AuNPs. In contrast, the addition of active Fe(II)⋅BLM retained the red color, probably because of the formation of protruding structures at the outermost phase of dsDNA‐AuNPs caused by BLM‐mediated DNA cleavage. Furthermore, the results of our model experiments indicate that oxidative base release and DNA‐cleavage pathways could be visually distinguished with color change. The present methodology was also applicable to model screening assays using several drugs with different mechanisms related to antitumor activity. These results strongly suggest that this assay with a rapid color change could lead to simple and efficient screening of potent antitumor agents.

## Graphical abstract

**The colorimetric detection** of bleomycin (BLM)‐mediated DNA cleavage with sequence selectivity is successfully demonstrated using dsDNA‐AuNPs with high colloidal stability. Notably, the cleavage pathways between oxidative base release and DNA cleavage can be identified according to a change in color using a colloidal stability test applied to dsDNA‐AuNPs with a blunt end and dangling end.

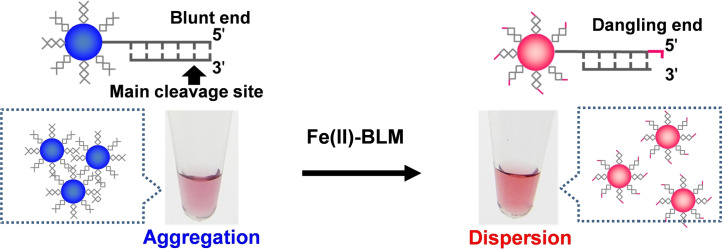

## 1 Introduction

**Figure 1:**
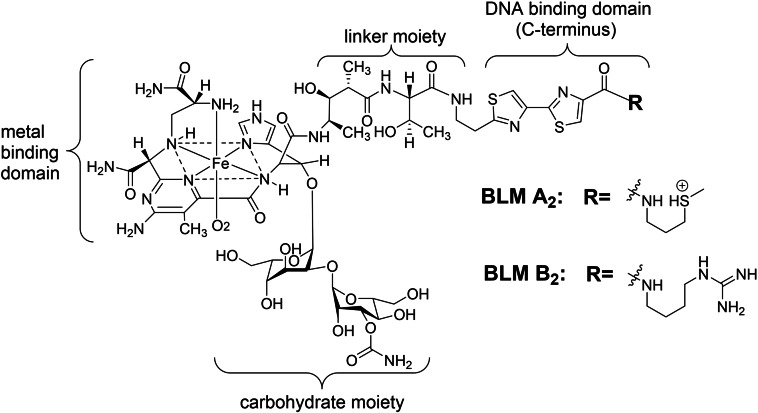
Chemical structure of clinically used bleomycin (BLM) with iron (II) in a mixture of BLM A_2_ and B_2_.

Bleomycins (BLMs), which are glycopeptides with a molecular weight of 1,500 Da, were originally isolated from *Streptomyces verticillus* and clinically used for chemotherapy of malignant lymphoma and squamous cell carcinoma.[^1^, ^2^] DNA damage reaction mediated by BLM is involved in the mechanism underlying its antitumor action. This shows that BLM chelated with iron (II) (Fe (II)⋅BLM) activates oxygen, which cleaves the 5′‐GC‐3′ and 5′‐GT‐3′ sequences of the DNA strand, resulting in proliferation suppression.[^3^, ^4^, ^5^, ^6^] The chemical reaction underlying DNA cleavage consists of (i) an electrostatic association with DNA, (ii) intercalation between DNA base pairs, and (iii) chemical cleavage action against the DNA strand (Figure 1).[^6^, ^7^] Consequently, a series of DNA cleavage events are achieved by linking BLM domains.

These BLM functions can be easily altered via the molecular design of BLM‐based structures with combinatorial synthesis. This includes the availability of the BLM gene cluster for biosynthesis^[8]^ for improved therapeutic efficacy as antitumor agents, allowing tumor targeting and cellular uptake[^9^, ^10^, ^11^] as well as multi‐gram scale synthesis of disaccharide moiety.^[12]^ In general, cleavage activity by BLM is evaluated using gel electrophoresis based on substrate ^32^P‐labeled DNA,[^13^, ^14^, ^15^] high‐performance liquid chromatography (HPLC),[^16^, ^17^, ^18^] and Förster resonance energy transfer[^19^, ^20^, ^21^, ^22^, ^23^, ^24^, ^25^, ^26^] including label‐free fluorescence method.^[27]^ Although these methods are important and useful for quantitative analysis of cleavage activity by BLM, they are time‐consuming and demand complex pre‐treatment steps during sample preparation with analog synthesis. Therefore, the present study focused on developing a visual method of evaluating BLM‐mediated DNA cleavage without labeling DNA substrate and without using specialized equipment.

Mirkin *et al*. reported that gold nanoparticles functionalized with single‐stranded (ss) DNA (ssDNA‐AuNP), through S‐Au interactions show unique colloidal properties not expressed in free‐form DNA.^[28]^ Furthermore, negatively charged and densely packed DNA layers can (i) improve the dispersibility of the nanoparticles, (ii) increase the relatively high DNA binding constant, and (iii) avoid enzymatic degradation.^[29]^ These attractive properties of ssDNA‐AuNPs allow them to develop crosslinked aggregation triggered by DNA duplex formation between ssDNA‐AuNPs, resulting in rapid color change owing to their unique optical properties originating from the size‐dependent excitation of the surface plasmon resonance (SPR) shift of AuNPs.[^30^, ^31^, ^32^] Therefore, this technology has been applied for colorimetric detection of biomolecules,[^29^, ^32^] nanoflare using gold nanoparticles as a quencher,[^34^, ^35^, ^36^] fabrication of superlattice nanostructures,[^37^, ^38^] and drug delivery systems.[^39^, ^40^, ^41^, ^42^] Maeda *et al*. also reported a non‐crosslinking aggregation occurrence that depends on the terminal base pairing of double‐stranded DNA (dsDNA) under high ionic conditions.^[43]^ dsDNA‐AuNPs with a full‐match sequence can undergo aggregation in ionic aqueous solutions, showing a drastic color change. In contrast, dsDNA‐AuNPs with a terminal mismatch sequence remain dispersed under identical conditions and are applicable for several bioassays, including ATP,^[44]^ single‐base substitutions,^[45]^ metal ions,[^46^, ^47^] liquid foods,^[48]^ and reversible control of interparticle distance on ssDNA‐templated dsDNA‐AuNP oligomers.[^49^, ^50^, ^51^, ^52^, ^53^] Such behavior may be due to differences between the attractive blunt‐end stacking at the matched dsDNA terminal and the repulsive mismatched dsDNA terminal on the particle. In fact, the analysis of the force‐distance curve between the dsDNA layers in atomic force microscopy supported the assumption.[^54^, ^55^] It was also found that single‐base protrusions on dsDNA‐AuNPs exhibited high colloidal stability even in high ionic aqueous solutions.^[56]^ The high colloidal stability with a dangling end on dsDNA‐AuNPs allowed more reliable human‐related cytochrome P450 *2C19* gene SNP genotyping, which plays a role in the metabolism of pharmaceutical agents.^[57]^ Consequently, a drastic color change can be controlled by a dangling end in the dsDNA located on the outermost surfaces of AuNPs, providing a new method to detect the colorimetric potent analytes among bioanalyses.

In this study, we developed a colorimetric method for detecting BLM‐mediated DNA cleavage to facilitate the selection of potent antitumor BLM analogs. The method is based on the high colloidal stability of the dsDNA‐AuNPs containing the main cleavage site for BLM. By using this method, we also identified the cleavage pathway via C4′‐hydroxy and C4′‐hydroperoxy intermediates. Furthermore, model colorimetric screening assays using several DNA‐associated drugs with different mechanisms were investigated.

## Experimental Section

### Materials

A colloidal dispersion of AuNPs with a nominal diameter of 15 nm was purchased from BBI Solutions (Cardiff, UK). The concentration of AuNPs was determined using the molar extinction coefficient (3.79×10^8^ L ⋅ mol^−1^ ⋅ cm^−1^ at 520 nm).^[56]^ Tris (2‐carboxyethyl) phosphine hydrochloride was purchased from Thermo Fisher Scientific (Waltham, MA, USA). Blenoxane (a mixture of BLM A_2_ and B_2_) and BLM A_5_ were purchased from AdooQ Bioscience (Irvine, CA, USA). The BLM concentrations were calculated using a molar extinction coefficient of 14,500 L ⋅ mol^−1^ ⋅ cm^−1^ at 292 nm in H_2_O.^[22]^ Cyclophosphamide (CPA), mitomycin C (MMC), cisplatin (CDDP), carboplatin (CBDCA), echinomycin (ECM), and metronidazole (MTZ) were purchased from FUJIFILM Wako Pure Chemical Corporation (Osaka, Japan). PCR microtubes (0.2 mL) for BLM‐mediated DNA cleavage were purchased from Nippon Genetics (Nippon Genetics Co., Ltd. Tokyo, Japan). Microplates (square solid‐bottom wells) for the model colorimetric assay with several DNA‐associated drugs were purchased from Aurora Microplates (Scottsdale, MT, USA). Deionized water (>18.1 MΩ ⋅ cm) purified with a Milli‐Q instrument (Millipore, Billerica, MA, USA) was sterilized before use in all experiments. Unmodified ssDNA and 3′‐mercaptopropyl 18‐nucleotide (nt) ssDNA (ssDNA‐SH) were purchased from Tsukuba Oligo Service (Ibaraki, Japan). All other reagents were purchased from FUJIFILM Wako Pure Chemical Corporation. The DNA concentration was determined by measuring the absorbance at 260 nm using a V‐630 UV/Vis spectrophotometer (JASCO Corporation, Tokyo, Japan). All ssDNA extinction coefficients for concentration determination were calculated using a data sheet provided by the manufacturer. The abbreviations and ssDNA sequences were as follows:

ssDNA‐SH: 5′‐CGCTTTTTTTTTTTTTTT‐SH‐3′,

complementary 18‐nt ssDNA: 5′‐AAAAAAAAAAAAAAAGCG‐3′,

complementary 18‐nt ssDNA with an abasic site: 5′‐AAAAAAAAAAAAAAAG_G‐3′,

complementary 16‐nt ssDNA for forming dangling end: 5′‐AAAAAAAAAAAAAAAG‐3′,

ssDNA‐SH without a main cleavage site: 5′‐AAATTTTTTTTTTTTTTT‐SH‐3′, and

complementary 18‐nt ssDNA without a main cleavage site: 5′‐AAAAAAAAAAAAAAATTT‐3′.

### ssDNA‐AuNP preparation

ssDNA‐AuNPs were prepared according to the literature.[^43^, ^56^] First, a stock solution of 50 μL of 100 mmol ⋅ L^−1^ ssDNA‐SH with or without the cleavage sequence of BLM in water, purified by an ethanolic precipitation procedure, was mixed with AuNPs. The mixture was then incubated at 50 °C for 1 d, followed by adding phosphate buffer (PB) solution (0.5 mol ⋅ L^−1^) and NaCl (2.5 mol ⋅ L^−1^) to obtain a final concentration of 10 mmol ⋅ L^−1^ PB solution containing 100 mmol ⋅ L^−1^ NaCl. Next, the buffer solution was incubated at 50 °C for 2 d and purified via centrifugation at 18,700×*g* at 10 °C for 25 min to obtain ssDNA‐AuNP. The ssDNA‐AuNP concentration was estimated using a molar extinction coefficient (4.10×10^8^ L ⋅ mol^−1^ ⋅ cm^−1^ at 525 nm). The final dsDNA‐AuNP concentration for the colorimetric assay was set at 5 nmol ⋅ L^−1^. PB solution (10 mmol ⋅ L^−1^, pH 7.4) containing 100 mmol ⋅ L^−1^ NaCl was used as the dispersion medium.

### Determination of ssDNA numbers on ssDNA‐AuNP

A stock solution of 60 μL of 10 nmol ⋅ L^−1^ ssDNA‐AuNP was treated with 60 μL of 1 mol ⋅ L^−1^ DTT in an aqueous solution to release the ssDNA‐SH on the AuNP surface. The mixture was incubated at 20–25 °C overnight. Next, after removing the AuNPs via centrifugation at 18,700×*g* for 25 min, the released probe in the supernatant was quantified using the OliGreen ssDNA Quantitation Kit (Molecular Probes, Thermo Fisher, MA, USA). The experiment was performed in triplicate (n=3).

### Colorimetric BLM‐mediated dsDNA‐AuNP cleavage assay

A stock solution of 5 μL of 20 nmol ⋅ L^−1^ ssDNA‐AuNP in 10 mmol ⋅ L^−1^ PB (pH 7.0) containing 100 mmol ⋅ L^−1^ NaCl was added to a solution containing 1 μL of 1 % Tween 20 and 2.5 μL of 4 μmol ⋅ L^−1^ complementary ssDNA to form dsDNA‐AuNP. Next, 2.5 μL of 80 μmol ⋅ L^−1^ BLM was added to this mixture, followed by incubation at 20–25 °C for 10 min. Next, 5 μL of 40 μmol ⋅ L^−1^ Fe(NH_4_)_2_(SO_4_)_4_ was added to the solution. Afterwards, 5 μL of water was added to the control sample without Fe^2+^, and the mixture was incubated at 20–25 °C for 20 min. Finally, 4 μL of 2.5 mol ⋅ L^−1^ NaCl was added to induce non‐crosslinking dsDNA‐AuNP aggregation (final volume: 20 μL/batch). The final concentrations of dsDNA‐AuNP, NaCl, and Fe(II)⋅BLM were 5, 0.5, and 10 μmol ⋅ L^−1^, respectively. These procedures were repeated for the colorimetric assay to determine Fe(II)⋅BLM concentration dependency ranging between 0–10 μmol ⋅ L^−1^ and for screening the BLM model with several DNA‐associated drugs (62 μmol ⋅ L^−1^ final concentration).

To examine the colorimetric cleavage pathway, a 20 % feed ratio was provided by mixing complementary 16‐nt and 18‐nt DNA, followed by the addition of ssDNA‐AuNP to form a 20 % dangling end on the outermost surface of dsDNA‐AuNP. Finally, NaCl was added to induce non‐crosslinking dsDNA‐AuNP aggregation (final volume: 20 μL). The final concentrations of dsDNA‐AuNP and NaCl were 5 nmol ⋅ L^−1^ and 0.5 mol ⋅ L^−1^, respectively.

### Kinetic assay of BLM‐mediated dsDNA‐AuNP aggregation

dsDNA‐AuNP aggregation via BLM‐mediated DNA cleavage was evaluated using a total volume of 100 μL and a procedure similar to that described above. A solution containing dsDNA‐AuNPs (final concentration: 5 nmol ⋅ L^−1^) and activated Fe(II)⋅BLM (final concentration: 2–10 μmol ⋅ L^−1^) was incubated for 30 min at 20–25 °C, followed by the addition of NaCl (final concentration: 0.5 mol ⋅ L^−1^) to evaluate the time‐dependent colloidal stability of dsDNA‐AuNPs. The changes in the UV‐vis absorption spectra were evaluated using the ratio of the absorbance at 530 nm and 700 nm (A_530_/A_700_).

### Transmission electron microscopy (TEM)

For sample preparation, 1 μL of dsDNA‐AuNPs (5 nmol ⋅ L^−1^) in 2.5 mmol ⋅ L^−1^ PB solution with 0.5 mol ⋅ L^−1^ NaCl was dropped onto an elastic‐carbon‐coated copper grid (ELS‐C10, Okenshoji, Tokyo, Japan) followed by removal of the excess solution by blotting with a filter paper. The measurements were performed using TEM (JEM 1230 microscope, JEOL, Tokyo, Japan) operated at an accelerating voltage of 80 kV.

## 2 Results and Discussion

**Figure 2:**
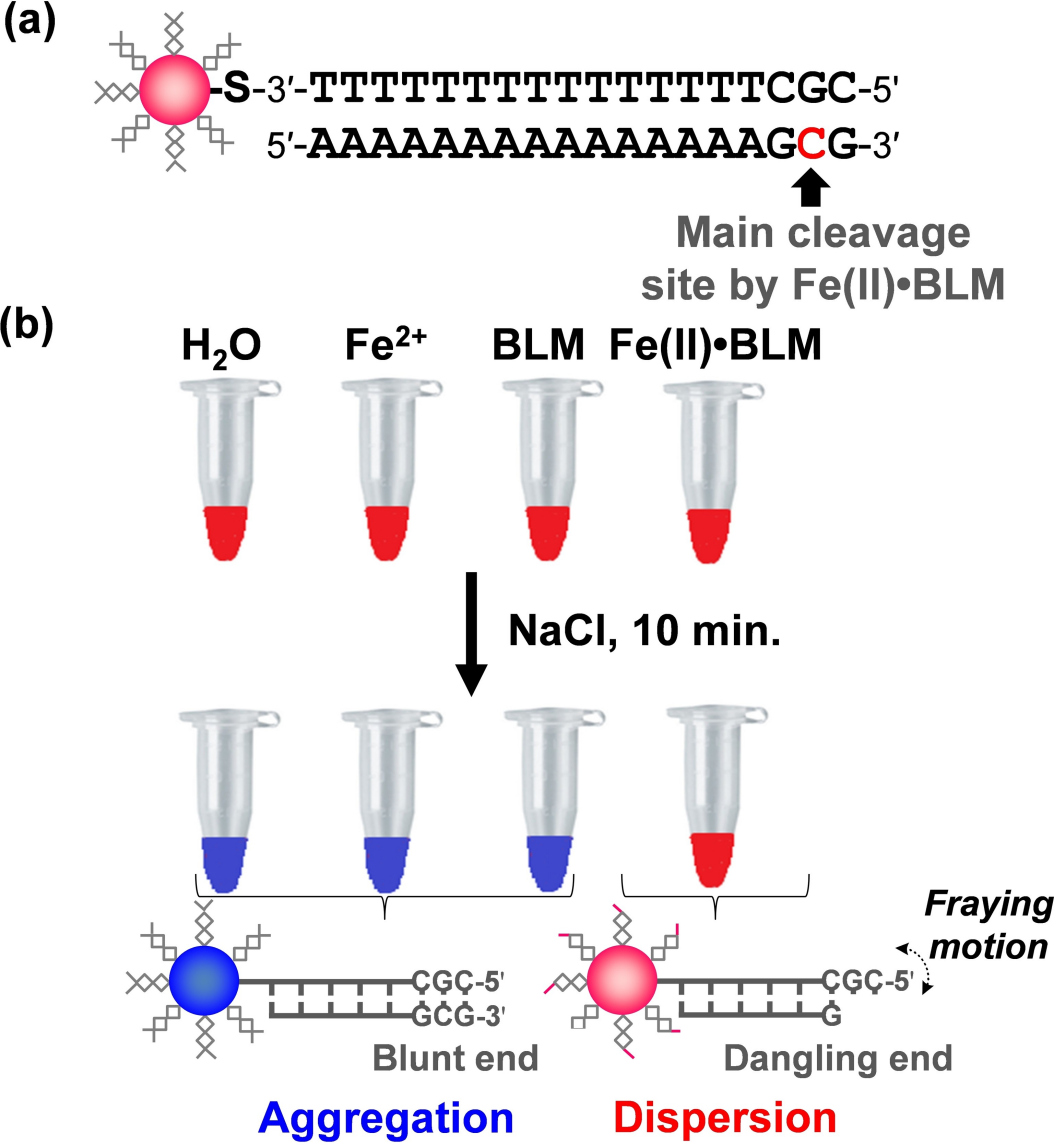
a) Structure of dsDNA‐AuNP having a main cleavage site of BLM. b) Schematic illustration of colorimetric BLM‐mediated DNA cleavage on dsDNA‐AuNPs having a cleavage site of Fe(II)⋅BLM.

To achieve a colorimetric method to detect BLM‐mediated DNA cleavage, we focused on DNA substrates for BLM‐mediated DNA cleavage. Hecht *et al*. reported a 16‐nt hairpin DNA (5′‐CG **C**_3_TTTAAAAAAAG **C**_15_G‐3′) for evaluation of BLM analog activities.^[22]^ The important point is that treatment with Fe(II)⋅BLM can induce a major cleavage at cytidine_15_ (not at cytidine_3_), allowing us to design the base sequence of dsDNA substrate containing a major cleavage site (5′‐GC‐3′) on the polyA strand. Consequently, 5′‐CGC‐3′ was chosen for 18‐nt 3′‐thiolated ssDNA with TA base pairs; it was attached to AuNPs with a 15‐nm diameter and mixed with 18‐nt complementary ssDNA to form dsDNA on AuNP (dsDNA‐AuNP) (Figure 2a). This design indicated that the BLM‐mediated DNA cleavage at the 5′‐GC‐3′ site on the polyT strand would be negligible because the dsDNA substrate has a major cleavage site on the polyA strand, thus expecting a simple and valuable *in vitro* evaluation method for BLM‐induced cleavage activity. The dsDNA‐AuNPs were initially treated with a high concentration of Fe(II)⋅BLM containing a mixture of BLM A_2_ and BLM B_2_ (final concentration: 62 μmol ⋅ L^−1^), followed by the addition of NaCl to induce non‐crosslinking aggregation.^[43]^ The mixture was incubated for 10 min at 20–25 °C to visually evaluate color changes (Figure 2b). Control samples, namely H_2_O, Fe^2+^, or BLM, were adapted using the same procedure as those for Fe(II)⋅BLM.

**Figure 3:**
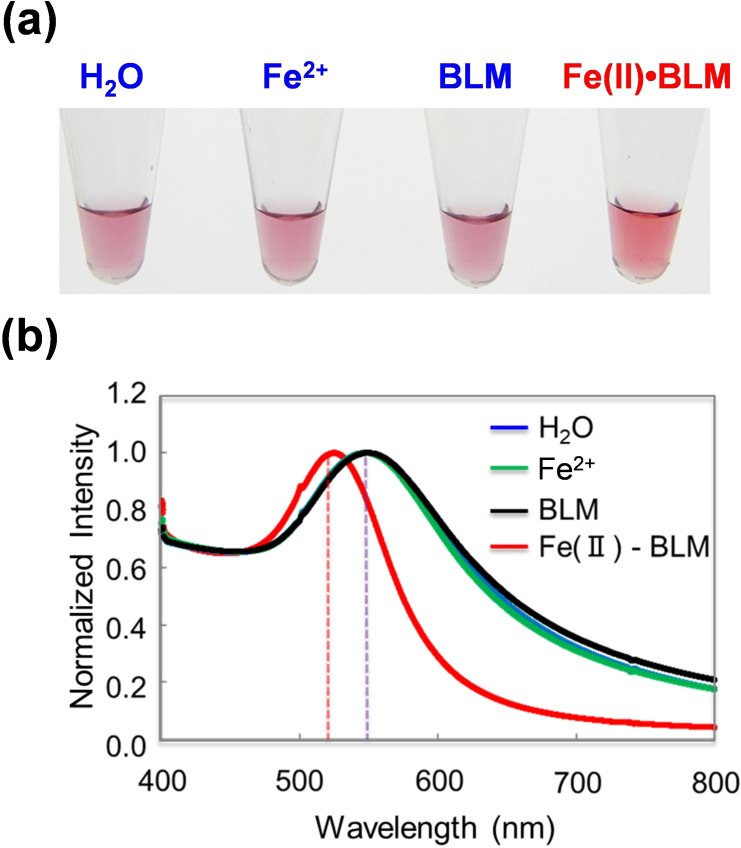
a) dsDNA‐AuNP treated with 62 μmol ⋅ L^−1^ of Fe(II)‐BLM compared with H_2_O, Fe^2+^, and BLM. (b) UV‐Vis spectra of dsDNA‐AuNP treated with 62 μmol ⋅ L^−1^ of Fe(II)⋅BLM compared with H_2_O, Fe^2+^, and BLM.

The dsDNA‐AuNP solution treated with Fe^2+^ or BLM showed rapid aggregation, resulting in a drastic color change from red to purple owed to a shift in SPR (Figure 3a). The tendency of color changes was similar in the sample with H_2_O. Thus, non‐crosslinking dsDNA‐AuNP aggregation inhibition owing to random cleavage by Fe^2+^ or BLM binding to DNA should be negligible. Contrarily, BLM activated with Fe(NH_4_)_2_(SO_4_)_2_ (Fe(II)⋅BLM) retained the red color. In addition, treatment of dsDNA‐AuNPs with Fe(II)⋅BLM resulted in change in the optical properties of the dsDNA‐AuNP dispersion (*λ*_max_=525 nm), as revealed via UV‐vis absorption spectroscopy. Treatment of dsDNA‐AuNP with several controls (H_2_O, Fe^2+^, or BLM) exhibited a red shift in the characteristic SPR (*λ*_max_=548 nm) and an increase in the intensity at approximately 700 nm related to aggregation state (Figure 3b). These spectral changes can be accompanied by a corresponding change from red to purple in the solution. This is probably due to the fact that Fe(II)⋅BLM treatment causes DNA cleavage at the outermost phase of dsDNA‐AuNP, and the protruding structure at the terminal in dsDNA‐AuNPs inhibits the attractive blunt‐end stacking formed between dsDNA‐AuNPs. In addition, when the Fe(II)⋅BLM concentration was reduced, a solution of dsDNA‐AuNP containing 10 μmol ⋅ L^−1^ BLM or Fe(II)⋅BLM also exhibited apparent colorimetric and spectroscopic changes between BLM and Fe(II)⋅BLM (Figure S1).

Furthermore, dsDNA with 18 A‐T base pairs (without main cleavage site) was attached to AuNPs (AT‐dsDNA‐AuNPs) using the same procedure for dsDNA‐AuNP with a main cleavage site. The colloidal stability of AT‐dsDNA‐AuNP thus obtained was evaluated by salt concentration dependency of color changes in comparison to dsDNA‐AuNP. In the case of dsDNA‐AuNPs, Fe(II)⋅BLM and BLM treatment gave clear color changes in the salt concentration range of 0.4 to 0.7 mol ⋅ L^−1^ (Figure S2‐(a)). On the other hand, both Fe(II)⋅BLM and BLM at 10 μmol ⋅ L^−1^ in AT‐dsDNA‐AuNP did not produce a color change at the salt concentration ranging from 0.4 to 0.7 mol ⋅ L^−1^ (Figure S2‐(b)). Additionally, a higher salt concentration of 0.7 mol ⋅ L^−1^ in non‐crosslinking AT‐dsDNA‐AuNP aggregation by BLM than that of the G‐C base pairs in dsDNA‐AuNP was required, presumably because of differences in number of hydrogen bond between the A‐T and G‐C base pairs.^[58]^ Therefore, AT‐dsDNA‐AuNP aggregation after treatment with Fe(II)⋅BLM suggests that non‐specific DNA cleavage by Fe(II)⋅BLM is negligible. A colorimetric assay of BLM bound to free AuNPs without modifications to the surface has been developed.^[59]^ However, the principle underlying the color change is the aggregation of free AuNPs induced by electrostatic neutralization between positively charged BLM and negatively charged AuNPs. As this color change does not corresponds to DNA cleavage, our data is the first report of naked‐eye detection of DNA‐cleavage ability of BLM derivatives, to the best of our knowledge.

**Figure 4:**
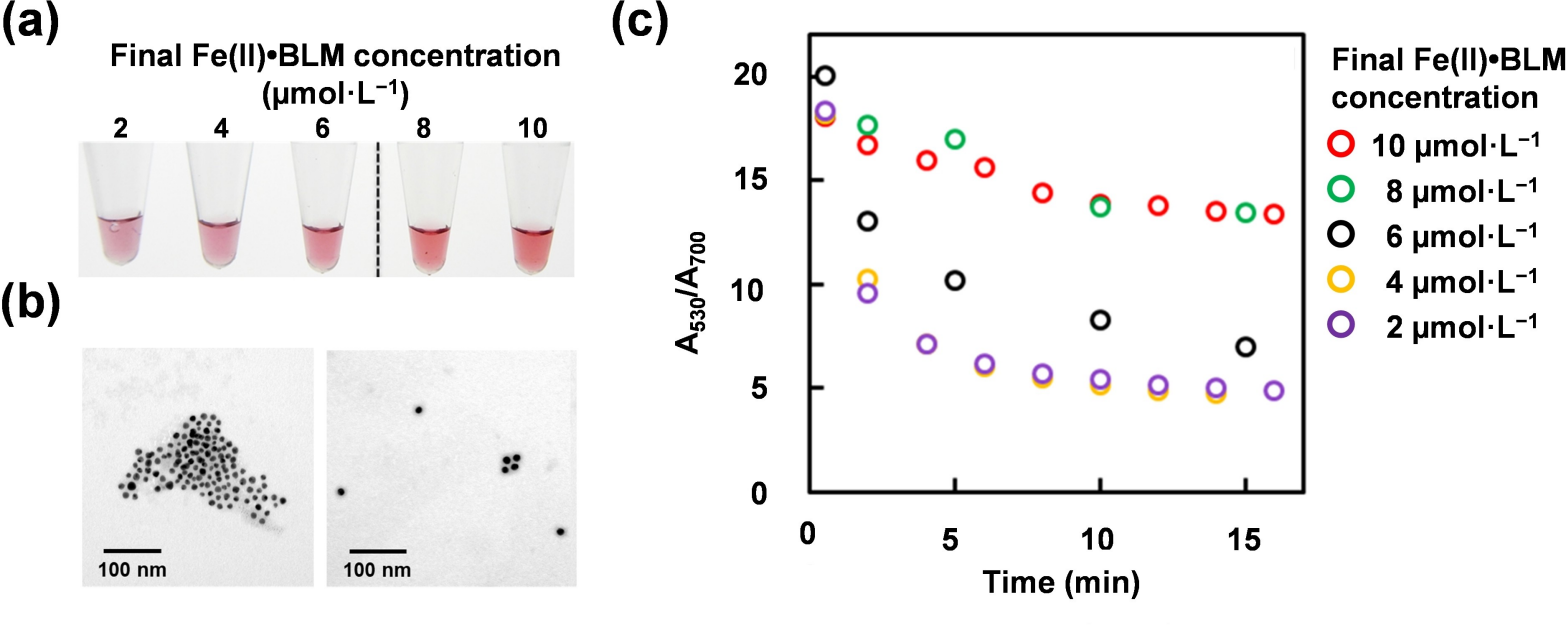
a) Dependency of Fe(II)⋅BLM concentration ranging from 2 to 10 μmol ⋅ L^−1^. b) TEM images of dsDNA‐AuNPs treated with (left panel) 2 μmol ⋅ L^−1^ and (right panel) 10 μmol ⋅ L^−1^ of Fe(II)⋅BLM. c) Time course of the change in the ratio of the absorbance at 530 nm and 700 nm for dsDNA‐AuNP aggregation induced by Fe(II)⋅BLM in the concentration range of 2 to 10 μmol ⋅ L^−1^.

BLM concentration dependency was attempted using a solution containing different concentrations of Fe(II)⋅BLM (2–10 μmol ⋅ L^−1^) in dsDNA‐AuNP solutions. Solutions with Fe(II)⋅BLM concentrations higher than 8 μmol ⋅ L^−1^ retained the red color, in contrast to those of lower concentrations, ranging from 2–6 μmol ⋅ L^−1^ (Figure 4a). In our previous paper, we reported that the colloidal stability of dsDNA‐AuNP depended on the feed ratio of the protruding structures on the particles; when the feed ratio of the single nucleotide protrusion exceeded 10 %, the colloidal dispersion showed a red color at 0.5 mol ⋅ L^−1^ of NaCl.^[56]^ This result indicate that the red color (dispersed state) of the dsDNA‐AuNP solution treated with 8 μmol ⋅ L^−1^ Fe(II)⋅BLM was maintained because of the appearance of a protruding structure of at least 10 % on the particles. Furthermore, the results of Fe(II)⋅BLM, regarding the aggregated state at 2 μmol ⋅ L^−1^ (left panel) and dispersed state at 10 μmol ⋅ L^−1^ (right panel), were supported by TEM images (Figure 4b).

To evaluate the time dependency of particle aggregation with concentration‐dependent behavior, the ratio of the intensity of absorbance derived from dispersion of AuNPs at 530 nm to absorbance derived from the aggregation of AuNPs at 700 nm (A_530_/A_700_) was adapted.^[60]^ The A_530_/A_700_ value of all samples, at all concentration ranges was 20, which is consistent with results of a previous study.^[60]^ The A_530_/A_700_ value of the solution treated with 8–10 μmol ⋅ L^−1^ of Fe(II)⋅BLM was maintained at approximately 70 % (A_530_/A_700_=14) after 15 min and retained a red color (green and red circles in Figure 4c). In contrast, the A_530_/A_700_ value in 2–4 μmol ⋅ L^−1^ of Fe(II)⋅BLM decreased to 40 % (A_530_/A_700_=5.0) in 5 min (purple and yellow circles in Figure 4c). The rate of non‐crosslinking dsDNA‐AuNP aggregation appeared to be significantly faster than that of crosslinking dsDNA‐AuNP aggregation, which occurred during 20–30 min for a half of the A_530_/A_700_ value in dsDNA‐AuNP aggregation induced by 300 nmol ⋅ L^−1^ of 13‐nt full‐match crosslinking ssDNA.^[60]^ The rate of non‐crosslinking and crosslinking aggregation depends on the ratio of gold to DNA. The ssDNA‐AuNP used in this study carried approximately 150 ssDNA molecules per particle, and the molar ratio of AuNP to ssDNA was approximately 0.75 equivalent. Previous data showed that non‐crosslinking aggregation, at around 1 equivalent of complementary DNA, occurred in regions where non‐crosslinking aggregation is faster than crosslinking aggregation.^[61]^ This tendency indicates the possibility of choosing a better selection method that uses faster non‐crosslinking aggregation for colorimetric BLM‐mediated DNA‐cleavage assays. In addition, for spectroscopic evaluation, the limit of detection to identify the color change was 6 μmol ⋅ L^−1^ (black circle in Figure 4c). Although this limitation value is approximately 10‐folds higher than that associated with fluorescent molecular beacon strategies,[^19^, ^22^] our results suggest the use of a practical concentration range and sufficient volume (20 μL/sample) to examine the DNA‐cleavage ability of BLM‐based functional molecules prepared from solid‐phase synthesis because a few milligrams of BLM analogs can be obtained via parallel solid‐phase synthesis.[^62^, ^63^]

**Figure 5:**
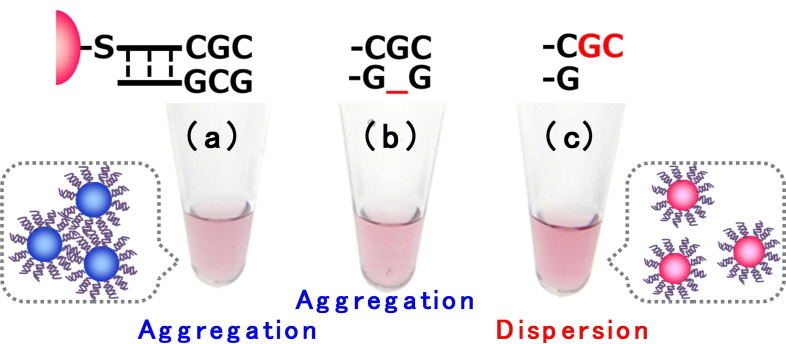
Colloidal stability of dsDNA‐AuNPs having duplexes assuming bleomycin‐mediated (a) no DNA damage, (b) DNA base release, and (c) DNA cleavage.

The next focus was the prediction of cleavage pathways using color change. To date, studies have been conducted on the BLM cleavage pathway using several DNA substrates. The two degradation pathways, oxidative base elimination through at C4′‐hydroxy intermediate, and oxidative DNA cleavage through C4′‐hydroperoxy intermediate, have been identified in addition to the quantification of each decomposed fragment via HPLC.^[22]^ Our colorimetric strategy is based on the control of colloidal stability, in which the principle of color change is derived from the protrusion structure versus blunt‐end structure on dsDNA‐AuNPs. The structure after BLM‐mediated DNA cleavage on dsDNA‐AuNPs might be the same as that resulting from oxidative DNA cleavage through C4′‐hydroperoxy intermediate. Thus, we hypothesized that progression along the degradation pathway could be visualized as a color change caused by constructing model particles. This indicates the occurrence of the two cleavage pathways–oxidative base elimination and DNA cleavage after sequence‐specific cleavage of AuNPs by BLM. To test this hypothesis, we prepared AuNPs that formed with a decomposition structure reflecting the two cleavage pathways. Each dsDNA‐AuNP solution (final concentration 5 nmol ⋅ L^−1^) was separately treated with NaCl (final concentration 0.5 mol ⋅ L^−1^) and incubated at room temperature for 10 min. The sample of dsDNA‐AuNPs having inactive and oxidative base release showed spontaneous aggregation (Figure 5a and b), resulting in a drastic color change from red to purple. Conversely, the dsDNA‐AuNPs with a dangling end with a 20 % feed ratio of two base‐protrusion structures exhibited red color (Figure 5c). Therefore, in this model experiment, the sample (Figure 5b) with the base release motif showed a color change associated with aggregation, similarly to the inactive sample (Figure 5a); the sample with the oxidative cleavage motif (Figure 5c) retained the red color derived from the dispersion. These results strongly suggest that the color change between dsDNA modified with AuNPs and Fe(II)⋅BLM proceeds in the oxidative DNA‐cleavage pathway rather than via base release reaction in the two cleavage pathways. It should be noted that identification of the cleavage pathway using conventional fluorescent molecular beacon strategies is limited because both cleavage pathway can release fluorescent fragments.[^19^, ^22^] Consequently, our proposed method can detect BLM‐mediated sequence‐specific DNA cleavage colorimetrically, as well as the cleavage pathway as a color change.

**Figure 6:**
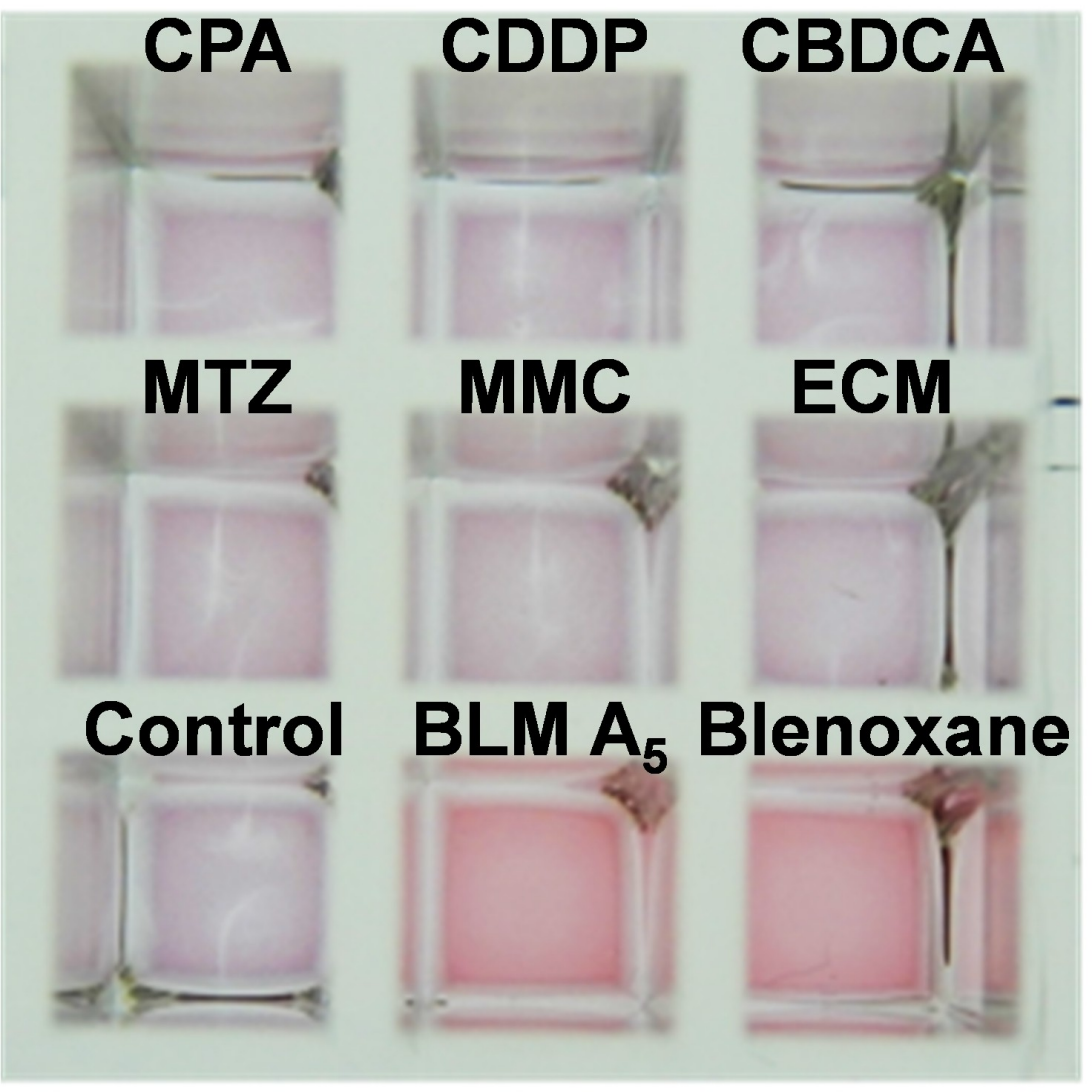
Model colorimetric assay of several DNA‐associated drugs including BLM A_5_ and Blenoxane (drug concentration: 62 μmol ⋅ L^−1^). CPA, cyclophosphamide; CDDP, cisplatin; CBDCA, carboplatin; MTZ, metronidazole; MMC, mitomycin C; ECM, echinomycin; Control, addition of H_2_O instead of drug.

After confirming that colorimetric BLM‐mediated DNA cleavage and the colorimetric cleavage pathway can work as expected under Fe(II)⋅BLM concentration dependency, we performed model colorimetric screening using several DNA‐associated agents with different medicinal mechanisms, as summarized in Table S1. CPA^[64]^ and MMC^[65]^ are nucleic acid alkylating agents; CDDP,^[66]^ CBDCA,^[67]^ and ECM^[68]^ are drugs that can be intercalated into the dsDNA strand; MTZ is a DNA‐damaging agent that induces the cleavage of 5′‐GT when converted to a nitroso compound by an intracellular redox reaction;^[69]^ as BLM analogs, BLM A_5_ ^[70]^ and clinically used BLM (mixture of BLMA_2_ and BLM B_2_),^[71]^ which contain different domains that electrostatically attract DNA, were used (Figure 1). As shown in Figure 6, the color observed in microplates containing each DNA‐associated drug (except BLM analogs) with dsDNA‐AuNPs changed from red to purple after 10 min of incubation. These alkylating agents and intercalators could stabilize dsDNA‐AuNPs against dispersion in an aqueous solution. However, our results showed that the alkylation of DNA to dsDNA‐AuNPs and the coexistence of intercalating DNA‐associated drugs did not prevent non‐crosslinking aggregation of dsDNA‐AuNPs. Conversely, both BLM A_5_ and clinically used BLM (Blenoxane), via the same mechanism of DNA cleavage, maintained the red color derived from the dispersed state. Therefore, we demonstrated the colorimetric screening of the activity of Fe(II)⋅BLM from sequence‐specific DNA cleavage of Fe(II)⋅BLM.

These results validate our dsDNA‐AuNP design. Notably, the cleavage sites in dsDNA introduced at the outermost surface of the particles could be essential for controlling colloidal stability with specificity against Fe(II)⋅BLM. If the strand with a 5′‐GC‐3′ site in the middle is adapted to dsDNA‐AuNPs, then (i) the presence of a dense DNA layer may introduce steric hindrance to the interaction between BLM and DNA, resulting in a loss of sequence properties; (ii) the duplex structure will be maintained with a nick even if BLM cleavage occurs. This may be attributed to the attractive blunt‐end stacking at the dsDNA terminal, resulting in the spontaneous non‐crosslinking aggregation of dsDNA‐AuNPs; and (iii) the translation of small structural changes corresponding to the cleavage pathway into the control of dsDNA‐AuNP dispersibility is difficult, as shown in our model experiment (Figure 5). This method controls the dispersion of dsDNA‐AuNPs with a protruding structure ratio of only a few percent, thus leading to a color change associated with BLM activity. Furthermore, it allows for the colorimetric discrimination of cleavage pathways. These characteristics represent a major technical difference relative to the crosslinking aggregation approach by using ssDNA‐AuNPs^[60]^ and molecular beacon strategy by using fluorescent hairpin DNA substrates.[^19^, ^22^]

## 3 Conclusion

We successfully demonstrated that dsDNA‐AuNPs could increase their colloidal stability by Fe(II)⋅BLM, and the reaction was concentration‐dependent. This concentration‐dependent behavior led to colorimetric changes, with 6 μmol ⋅ L‐1 being the detection limit for the BLM concentration. Furthermore, AT‐dsDNA‐AuNPs treated with BLM and Fe(II)⋅BLM exhibited the same color change tendencies resulting from spontaneous salt‐induced aggregation at the same salt concentration, suggesting sequence‐specific cleavage by Fe(II)⋅BLM could be detected colorimetrically. Consequently, we demonstrated the colorimetric detection of BLM‐mediated DNA cleavage using dsDNA‐AuNPs. More importantly, we demonstrated that the cleavage pathways between oxidative base release and DNA cleavage could be identified based on the change in color using a colloidal stability test in dsDNA‐AuNPs with abasic sites and protrusion structures of two bases. Experiments regarding the cleavage pathway for the characterization of decomposed fragments released from BLM‐mediated dsDNA‐AuNP cleavage according to mass analysis and the detailed kinetics will be published elsewhere. The methodology implemented could provide a practical non‐fluorescence‐based analysis method in which potent DNA‐damaging agents are colorimetrically screened, and sequence‐specific cleavage is more accurate based on combinatorial solid‐phase libraries.

## Conflict of interest

The authors declare no conflict of interest.

4

## Supporting information

As a service to our authors and readers, this journal provides supporting information supplied by the authors. Such materials are peer reviewed and may be re‐organized for online delivery, but are not copy‐edited or typeset. Technical support issues arising from supporting information (other than missing files) should be addressed to the authors.

Supporting Information

## Data Availability

The data that support the findings of this study are available on request from the corresponding author. The data are not publicly available due to privacy or ethical restrictions.
