# Supplementary material for: A Simple Colorimetric Assay of Bleomycin‐Mediated DNA Cleavage Utilizing Double‐Stranded DNA‐Modified Gold Nanoparticles
Source: Chembiochem. 2022 Oct 25;24(1):e202200451. doi: 10.1002/cbic.202200451 (PMC10092608; doi:10.1002/cbic.202200451)
Supplement: Supplementary file 1 — Supporting Information [file CBIC-24-0-s001.pdf]

ChemBioChem

Supporting Information

A Simple Colorimetric Assay of Bleomycin-Mediated DNA Cleavage Utilizing Double-Stranded DNA-Modified Gold Nanoparticles

Yoshitsugu Akiyama+,* Kazunori Kimura+, Syuuhei Komatsu, Tohru Takarada, Mizuo Maeda, and Akihiko Kikuchi

CONTENTS

1. UV-Vis spectroscopic characterization of dsDNA–AuNP stability………..............S-2

2. Colorimetric assay of dsDNA–AuNP with and without a cleavage site…………. . S-3

3. Proposed mechanism of BLM-mediated dsDNA–AuNP cleavage…………………S-4

4. Chemical structure of DNA-associated drugs..S-5

UV-Vis spectroscopic characterization of dsDNA–AuNP stability treated by BLM

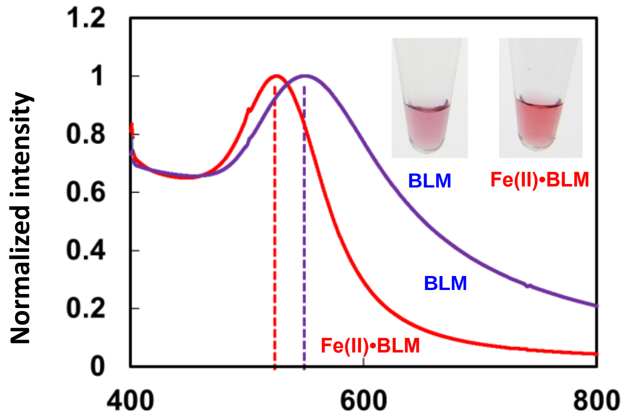

Figure S1. UV-Vis spectra of dsDNA–AuNP in the presence of 10 μmol· L^{-1} BLM and Fe(II)•BLM. Insert: Colorimetric assay of dsDNA–AuNP treated with 10 μmol· L^{-1} BLM (left) or Fe(II)•BLM (right).

Colorimetric assay of dsDNA–AuNP with and without a cleavage site

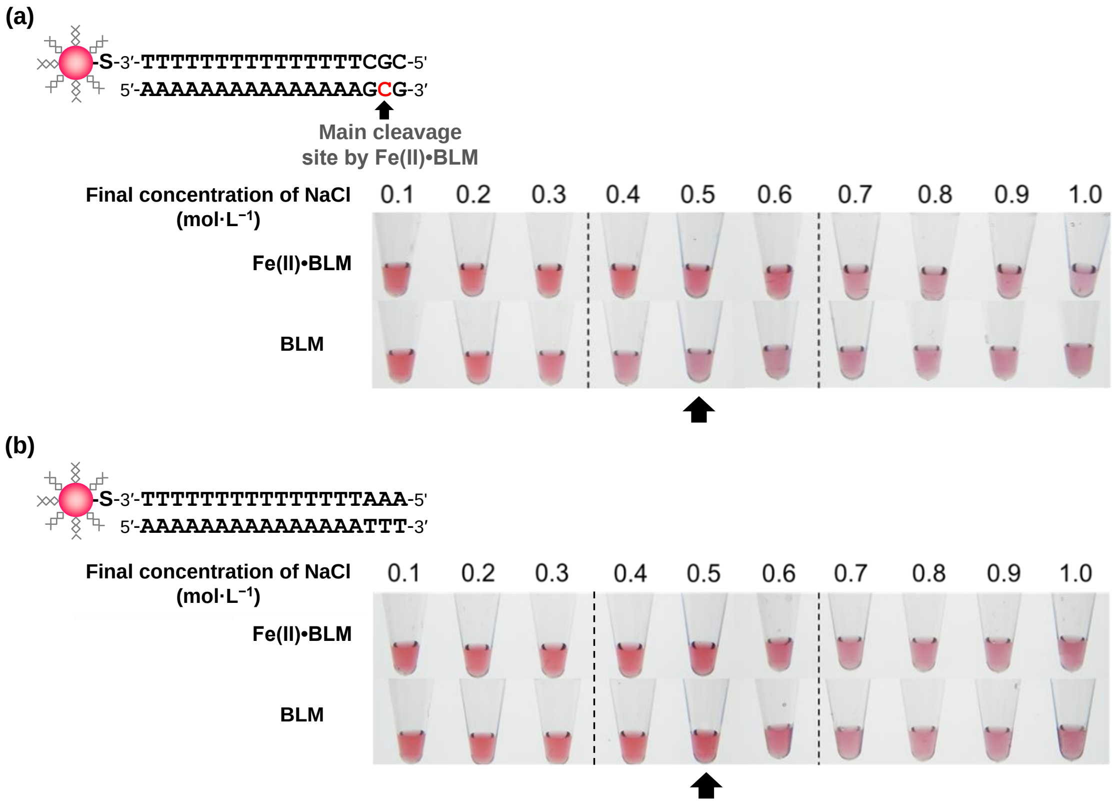

Figure S2. NaCl concentration dependency of color changes on dsDNA-AuNPs (a) with and (b) without a cleavage site by Fe(II)•BLM.

Proposed mechanism of BLM-mediated dsDNA–AuNP cleavage

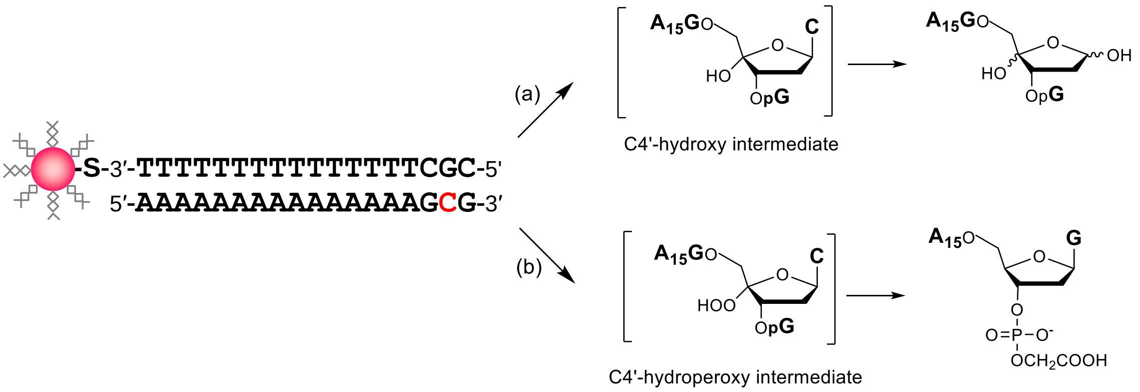

Figure S3. Proposed pathways of BLM-mediated dsDNA–AuNP damage: (a) oxidative base release via C4'-hydroxy intermediate and (b) oxidative cleavage via C4'-hydroperoxy intermediate.

Chemical structure of DNA-associated drugs

Table S1 Chemical structure of DNA-associated drugs for colorimetric assay

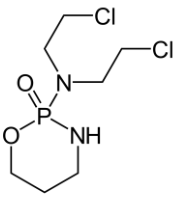	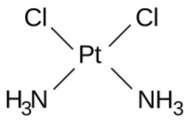	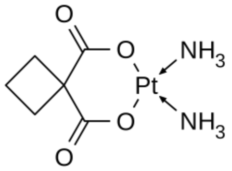
Cyclophosphamide (CPA)	Cisplatin(CDDP)	Carboplatin (CBDCA)
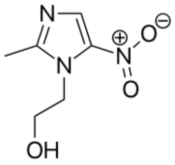	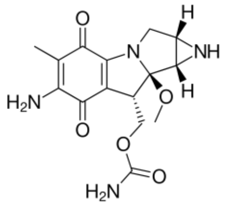	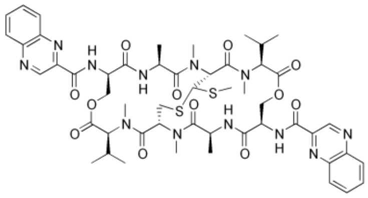
Metronidazole (MTZ)	Mitomycin C (MMC)	Echinomycin (ECM)
